# Advances in the Application of Aptamer Biosensors to the Detection of Aminoglycoside Antibiotics

**DOI:** 10.3390/antibiotics9110787

**Published:** 2020-11-07

**Authors:** Yunxia Luan, Nan Wang, Cheng Li, Xiaojun Guo, Anxiang Lu

**Affiliations:** 1 Beijing Research Center for Agricultural Standards and Testing, Agricultural Product Quality and Safety Risk Assessment Laboratory of the Department of Agriculture, Beijing Municipal Key Laboratory of Agriculture Environment Monitoring, Beijing 100097, China; luanyx@brcast.org.cn (Y.L.); wn18841617422@163.com (N.W.); lic@brcast.org.cn (C.L.); Guoxj@brcast.org.cn (X.G.); 2 College of Pharmacy, Jinzhou Medical University, Jinzhou 121001, China

**Keywords:** aptamer, aminoglycoside antibiotic, antibiotic detection, biosensors

## Abstract

Antibiotic abuse is becoming increasingly serious and the potential for harm to human health and the environment has aroused widespread social concern. Aminoglycoside antibiotics (AGs) are broad-spectrum antibiotics that have been widely used in clinical and animal medicine. Consequently, their residues are commonly found in animal-derived food items and the environment. A simple, rapid, and sensitive detection method for on-site screening and detection of AGs is urgently required. In recent years, with the development of molecular detection technology, nucleic acid aptamers have been successfully used as recognition molecules for the identification and detection of AGs in food and the environment. These aptamers have high affinities, selectivities, and specificities, are inexpensive, and can be produced with small batch-to-batch differences. This paper reviews the applications of aptamers for AG detection in colorimetric, fluorescent, chemiluminescent, surface plasmon resonance, and electrochemical sensors for the analysis in food and environmental samples. This study provides useful references for future research.

## 1. Introduction 

**Figure 1:**
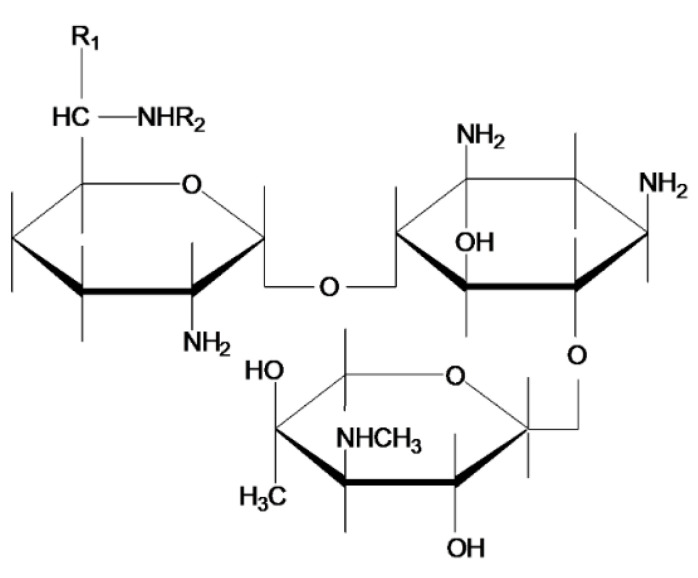
Chemical structure of aminoglycoside antibiotics.

Aminoglycoside antibiotics (AGs) are a class of broad-spectrum antibiotics whose molecules are composed of an aminocyclitol and one or more amino sugar molecules (d-glucosamine, d-kanosamine) connected by glucoside bonds (Figure 1) [1,2]. They are characterized by high polarity and hydrophilicity (logP values in the range from −4 to −9), and are very soluble in water, slightly soluble in methanol, and insoluble in non-polar organic solvents [3]. Most AGs, including kanamycin, neomycin, streptomycin, gentamicin, tobramycin, and spectinomycin, are biosynthetic and produced by different species of *Streptomyces* and *Micromonospora*; however, some, such as netilmicin, amikacin, and arbekacin, are semi-synthetic. As important antimicrobials, AGs kill bacteria mainly by inhibiting bacterial protein synthesis and destroying the integrity of the bacteria cell membrane. AGs are mainly used to treat various moderate and severe respiratory infections, urinary tract infections, intestinal infections [4], skin and soft tissue infections caused by Gram-negative bacteria, such as *Enterobacter*, *Klebsiella*, proteobacteria, *Pseudomonas aeruginosa*, and *Staphylococcus* [5,6,7,8]. Streptomycin and amikacin can be used for second-line treatment of tuberculosis [9,10]. Because AGs are inexpensive and have a good bacteriostatic effects [11], they are widely used in both clinical practice and in animal husbandry as veterinary drugs and growth factors. However, AGs have obvious side effects in the human body, such as nephrotoxicity and ototoxicity [12,13,14,15,16,17,18]. When an antibiotic is administered, some of the antibiotic is excreted and can cause environmental damage, while the rest remains in the body. After accumulation in the food chain, these residual antibiotics eventually enter the human body and can be very damaging to human health. Antibiotic abuse will accelerate the spread of antibiotic resistance and lead to decreased human immunity [19,20]. To date, China, the European Union, Japan, and the United States have established maximum residue limits (Table 1) for gentamicin, kanamycin, neomycin, streptomycin, and dihydrostreptomycin [21,22]. Therefore, a rapid, sensitive, and inexpensive method should be established for the detection of AG residues in food and the environment. The reported AGs assay methods include high performance liquid chromatography (LC) [23,24,25,26], LC-mass spectrometry (MS) [27,28,29,30,31,32,33,34], capillary electrophoresis-MS [35,36], microbial assays [37], and enzyme-linked immunoassays [38,39,40,41]. 

Because AGs are highly polar, they are poorly preserved in conventional reversed-phase LC, and they cannot be detected using ultraviolet or fluorescence detectors unless pre- or post-column derivatization is performed because they do not contain chromophores or fluorophores [42,43,44,45]. However, derivatization makes the instrument conditions more complex, and the additional processing steps required lead to loss of the analytical substance. Furthermore, excess reagents and derivatives can interfere with the results. LC-MS has been widely used for the analysis of AGs. However, different AGs are structurally similar and produces are similar ions after fragmentation, so quantitative determination of AGs is challenging. Additionally, the use of LC-MS for AG detection requires the use of volatile mobile phase additives, which is not beneficial for the long-term condition of the instrument. Consequently, pretreatment is needed when the sample is analyzed by an instrumental method. Because AGs are strongly polar and exist as polyanions in aqueous solutions, they are difficult to extract. Generally, solid-phase extraction is used to extract and purify AGs, but this method has a low recovery rate and the pretreatment steps are complex. Instrumental analysis has high sensitivity and accuracy, but it is expensive, requires trained operators, and is difficult to use for rapid on-site detection [46,47]. Microbial methods and enzyme-linked immunoassays are relatively simple but have long detection times and large experimental errors, which can result in interference and false positive for antibiotics with similar structures [48].

With recent developments in biotechnology, sensors based on specific biometric elements have been widely used for antibiotic residue detection [49]. Biosensors have strong specificities and high sensitivities, and are simple, small, and portable. The disadvantages of antibodies and enzymes as classical biosensors are that they have poor thermal stabilities, are difficult to modify, and have high production costs [50]. Antibody preparation usually requires immunization of animals and hybridoma techniques. AGs are small-molecule haptens and cannot stimulate the body to produce corresponding antibodies on their own, so it is difficult to obtain highly sensitive antibodies [21]. Aptamers as “chemical antibodies” are single-stranded deoxyribonucleic acid (ssDNA) or ribonucleic acid (RNA) sequences of approximately 10–100 bases that can specifically bind to a target [51]. They can be obtained from the nucleic acid molecular library through in vitro screening via the systematic evolution of ligands by exponential enrichment (SELEX) technique. As molecular recognition elements, aptamers can identify a wide range of target molecules, including metal ions, small drug molecules, proteins, viruses, animal cells, and tissues [52,53]. Although aptamers have many similar properties to antibodies, they have a number of advantages over traditional antibodies [54,55]. First, they have stronger specificities. Their affinities to target molecule are higher than those of antibodies, and their dissociation constants (*K*_d_) are far lower than those of antibodies and can be as low as nanomole to picomole per liter levels. Second, they can be used for a wider range of target molecules. Screening and synthesis studies have shown an aptamer can theoretically be selected for any target substance, whereas antibodies cannot. Third, aptamers are simpler to prepare than antibodies, and can be rapidly synthesized in large quantities in vitro. By contrast, antibodies need to be produced in an animal or cells, and the preparation conditions are relatively complex. Fourth, aptamers have good stabilities and are easy to store. Whereas antibodies are unstable and prone to irreversible degradation, aptamers are more stable and can recover their active conformations. Fifth, aptamers have low molecular weights and are easily chemically modified. Consequently, they can be coupled with various molecules by simple chemical reactions, which provides a convenient and flexible method for development of sensors that use different detection methods. Therefore, aptamers are ideal for molecular recognition with high specificity and affinity [56,57]. Additionally, aptamers can be used to introduce components for signal amplification, such as nanomaterials and fluorescent compounds, which greatly improves the detection sensitivity, shortens the detection time, and broadens the application prospects. Due to the unique optical, electrochemical, and mechanical properties of nanomaterials, such as metal nanoparticles, carbon nanotubes, nanocomposite, and nanostructuredmaterials, they have been widely used in aptasensing strategies. These nanomaterials are applied as catalytic tools, immobilization platforms, or as optical or electroactive labels in biosensor schemes in order to improve their performance [58]. Some AG aptamers are shown in Table 2.

## 2. Application of Aptamer Biosensors in the Detection of AGs

As a recognition element, aptamers do not directly generate detection signals. Therefore, researchers usually combine aptamers with nanoscale gold, quantum dots, or another material that can generate a photoelectric signal to form a composite probe. Aptamer biosensors are developed in combination with electrochemical and optical detection systems to realize the detection of target compounds. The operating modes for aptamer-based biosensors have been described in the reported review paper [63]. The differentially designed principles are necessary for different targets, as the differences in aptamer sequences and target characteristics. Previous reviews have focused on the classification of the perspective of compounds rather than the design of biosensors especially for AGs [63,64]. This review focuses on aptamer sensors (aptasensors) using colorimetric, fluorescent, chemiluminescent, surface plasma resonance (SPR), and electrochemical techniques for the detection of AGs. Table 3 summarizes different aptasensor methods and their detection performances. 

## 3. Colorimetric Aptamer Sensors

**Figure 2:**
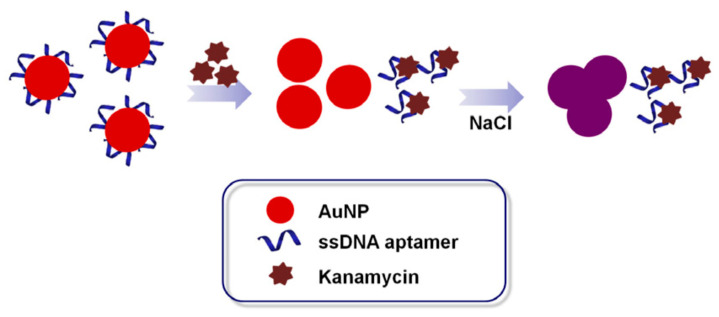
Schematic illustration of the AuNP-based colorimetric assay for detection of kanamycin [59].

The biggest advantage of the colorimetric method is that preliminary experimental results can be obtained easily by direct observation of color changes using a cell phone chromatism or spectrophotometer without the need for complex equipment [82]. Consequently, the colorimetric method is inexpensive, simple, and rapid [83,84,85]. Gold nanoparticles (AuNPs) are an excellent material for colorimetric aptasensors because they have unique optical properties, good biocompatibility, large surface areas, and high absorption efficiencies. Furthermore, AuNPs are also able to enhance other optical signals like fluorescence and light scattering [86,87,88,89]. AuNP-based aptamer colorimetric analysis has been widely used in kanamycin detection. Song [59] screened a kanamycin ssDNA aptamer, Ky2 (TGGGGGTTGAGGCTAAGCCGA), using the SELEX method, and established a AuNP-colorimetric method using this aptamer (Figure 2). In this method, an aqueous sodium chloride solution of dispersed AuNPs was wine red. In the presence of aptamers, the AuNPs coordinated with the aptamers via van der Waals attractions to maintain dispersion of the AuNPs in the solution. When kanamycin was added to the system, the aptamers bound to it specifically and more strongly than to the AuNPs. This resulted in dissociation of the aptamers from the AuNPs surface, aggregation of the AuNPs in the salt solution, a change in the solution color changed from red to blue or even purple, and a change in the absorbance. The detection limit of this method for kanamycin was 25 nmol/L. Compared with ssDNA, double stranded DNA does not protect AuNPs from salt-induced aggregation because of its rigid structure. Zhang [90] designed a colorimetric sensor using AuNPs and dsDNA to detect kanamycin. In the absence of kanamycin, the aptamer formed a stable DNA double strand with the complementary DNA strand, which resulted in salt aggregation of AuNPs. In the presence of kanamycin, the aptamer was released to bind to its target, and complementary DNA was adsorbed on the surfaces of the AuNPs to protect them from salt-induced aggregation. The absorbance ratio was linearly correlated to the concentration of kanamycin in the range of 0.02–0.3 mol/L, and the detection limit was 8 nmol/L. To extend on the classic AuNP colorimetric aptasensors, Chen et al. [91] introduced fluorescence labeling to develop a sensor with a dual signals for kanamycin A. The detection limit of this method reached 0.3 nmol/L, and it was successfully applied to the analysis of milk samples. Another colorimetric aptasensor has been developed using the catalyzed chromogenic reactions of various enzymes or mimic enzymes. Zhao [65] established a new method for streptomycin colorimetric detection using the simulated enzyme catalytic activity of AuNPs. When there was no streptomycin, the aptamer bound to the AuNPs, which reduced the activity of the AuNPs enzyme. In the presence of streptomycin, the aptamer could not bind to the AuNPs, and the enzyme activity of the AuNPs was observed. The detection limit of this method for streptomycin was 86 nmol/L and the linear range was 0.1–0.5 mol/L. Silver nanoparticles (AgNPs) have similar optical properties to AuNPs and also aggregate in the presence of a salt. Because the amino group in kanamycin can bind to AgNPs via a Ag-N bond, it can adsorb on the surface of unmodified AgNPs and prevent salt-induced aggregation. This protective mechanism will be weakened after kanamycin binds to an aptamer. According to the selection mechanism of the aptamer and AgNPs protection by the analyzed target, Xu [66] designed a new aptasensor. This method could detect kanamycin in milk samples at 0.05–0.6 μg/mL levels within 20 min with a detection limit of 2.6 ng/mL. Aptamer biosensors using AuNPs or AgNPs for colorimetric detection have strong specificities and high sensitivities, are simple to make and easy to use, and have been widely used in field and for label free detection. However, AuNP-based aptamer colorimetric analysis usually need large amount of aptamer, as the dispersion of the AuNPs and the binding of target all need certain amount of aptamer, so the sensitivity of colorimetric aptasensors should be more improved through powerful signal amplification methods.

## 4. Fluorescent Aptamer Sensors

**Figure 3:**
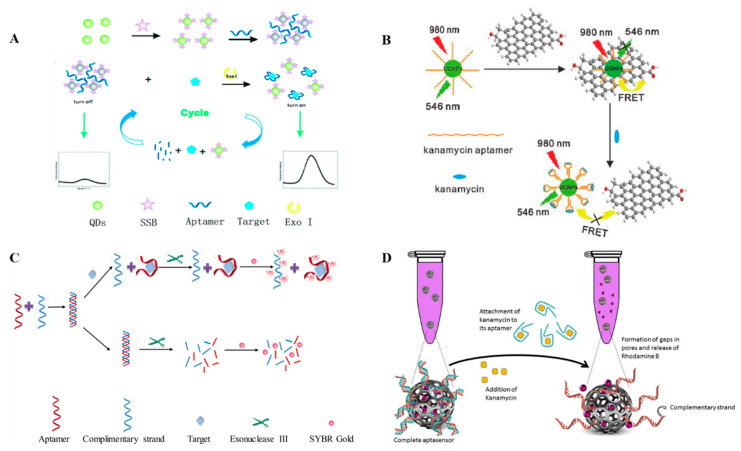
Schematic illustration of a fluorescent aptasensor. (**A**) A fluorescent “on” switch aptasensor containing QDs-SSB [69]. (**B**) Fluorescence resonance energy transfer between kanamycin aptamer UCNPs and graphene [70]. (**C**) Unlabeled fluorescence analysis [71]. (**D**) dsDNA-modified mesoporous silicon nanoparticles (MSNs) loaded with rhodamine B [72].

Fluorescence is a highly sensitive optical property. The effectiveness of aptasensors using fluorescent-labeled probes has been confirmed in many experimental studies. Sharma constructed an aptaswitch sensor complex using a combination of fluorophore and quenching labeled oligonucleotides and an aptamer that recognizes chloramphenicol [92,93]. Aptamer fluorescence detection uses the specific recognition of an aptamer and target antibiotics to regulate the energy transfer efficiency between a fluorescent donor and a recipient, to achieve quantitative detection of target antibiotics through changes in the fluorescence intensity or polarization [94,95]. According to the different modes of action of the fluorophore and aptamer, the sensors can be classed as labeled and unlabeled fluorescent aptasensors. To construct labeled fluorescent aptasensors, organic small molecule fluorescent dyes or fluorescent nanomaterials are usually used to label the sensor probes. Ramezani [67] designed a fluorescent aptasensor using a kanamycin aptamer complementary sequence labeled with exonuclease III, AuNPs, and carboxyfluoresce in FAM. This sensor was suitable for the detection of kanamycin residues in food with a detection limit as low as 321 pmol/L. Ling [68] divided the RNA aptamer of neomycin B into two segments, one of which was absorbed on the surfaces of AuNPs by polyadenylate, and the other labeled with the FAM fluorophore. When neomycin B was present in the samples, the target material and the two nucleic acid fragments were rapidly assembled into a compact H-shaped structure on the AuNPs surface, leading to quenching of the FAM fluorophore. The concentration of neomycin B in the solution was inversely proportional to the fluorescence intensity, and the detection limit for neomycin B was 0.01 mol/L. Because of the shortcomings of fluorescent dyes, such as poor photobleaching resistance and vulnerability of the fluorescence performance to external factors, some fluorescent nanomaterials with better performance have been applied to the construction of fluorescent aptasensors for AGs. Quantum dots (QDs) have attracted much attention because of their unique optical and electronic properties, including high luminescence, strong light stability, good resistance to light bleaching, wide absorption bands, and adjustable sizes [96,97]. Wu [69] designed a novel fluorescent switch sensor using QDs labeled with ssDNA binding protein (SSB) and exonuclease I for assisted target recovery, and applied it to streptomycin detection (Figure 3A). The fluorescent probes were synthesized by labeling QDs with SSB, which could bind to the aptamer specifically. When an aptamer was added as a bridge ligand, it hybridized with SSB. At the same time, the QDs scattered in the solution aggregated, which resulted in self-quenching, and the sensor state changed from “on” to “off”. In the presence of streptomycin and exonuclease I, the aptamer preferentially bound to the target. Exonuclease I then digested the aptamer target into a single nucleotide, and the released target could participate in the reaction cycle and produce a strong fluorescence signal. At this point, the distance between the QDs increased and the fluorescence intensity recovered. Thus, the switch changed from the “off” state to the “on” state. Under the optimum conditions, there was a good linear relationship between the fluorescence intensity and streptomycin concentration in the range of 0.1–100 ng/mL and the detection limit of this method was about 0.03 ng/mL. Li [70] used upconversion with nanoparticles and graphene oxide to develop an aptasensor using fluorescence resonant energy transfer technology for detection of kanamycin (Figure 3B). Under the optimized conditions, the method had a wide linear detection range (0.01–3 nmol/L), low detection limit (9 pmol/L), and showed good performance on application to real samples. Compared with a labeled fluorescent aptasensor, time-consuming probe labeling and purification steps were not required for construction of this unlabeled fluorescent aptasensor, which saved time and reduced inter-batch differences in the sensor preparation. Taqhdis [71] used exonuclease III, a fluorescent dye (SYBR Gold), and an aptamer complementary strand to establish an unlabeled fluorescence analysis method for detection of streptomycin in milk and blood samples (Figure 3C). Without streptomycin, the fluorescence intensity was weak. After adding streptomycin, the aptamer combined with the target, leading to release of the aptamer complementary strand, which protected against exonuclease III activity. With addition of SYBR Gold, a strong fluorescence signal was observed. The sensor had high selectivity for streptomycin, and the detection limit reached 54.5 nmol/L. Dehghani [72] constructed a double-stranded “molecular gate” closed mesoporous silicon probe by efficiently loading mesoporous silicon nanoparticles (MSNs) on small molecule fluorescent dyes, and developed a fluorescent aptasensor for detection of kanamycin without the need for a signal amplifier (Figure 3D). The amine-modified complementary chain was covalently fixed on the MSNs surface and the unlabeled aptamer was also fixed on the MSNs surface through pairing with the complementary chain. In the presence of kanamycin, the aptamer specifically bound to it and was separated from its complementary chain and the double-stranded “molecular gate” was destroyed, leading to release of rhodamine B. The fluorescence intensity of the solution increased with leakage of rhodamine B. Kanamycin was detected by measuring the fluorescence intensity. The linear range for measurements using the relative fluorescence intensity was 24.75–137.15 nmol/L and the detection limit was 7.5 nmol/L. Fluorometric sensing is more promising methodology to analyze and measure AGs quantitatively and sensitively compared to colorimetric assay. However, most of the fluorescent aptamer sensors could only detect one target in an assay, if more sensors could be designed using multicolor quantum dots, it would be helpful to achieve the efficient detection of multi-targets.

## 5. Chemiluminescent Aptamer Sensors

**Figure 4:**
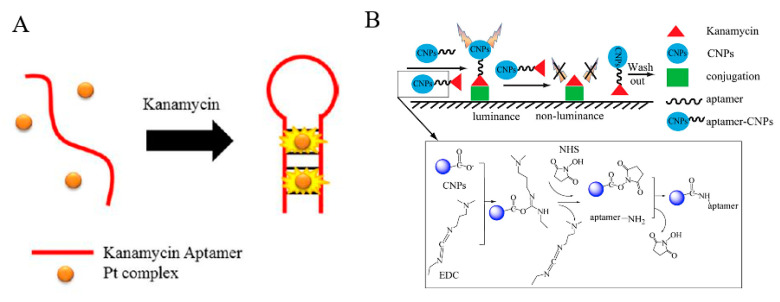
Schematic illustration of a chemiluminescent aptasensor. (**A**) A luminescent probe containing an aptamer and Pt(II) [73]. (**B**) A carbon nanoparticles aptasensor [74].

Chemiluminescence, also known as cold light, refers to light radiation produced by chemical reactions in the absence of any light, heat, or electric field excitation [98]. Because there is no need for an external excitation light source, interference from background or stray light is avoided, the level of noise is reduced, and the signal-to-noise ratio is greatly improved. Because of its high sensitivity, wide linear range, fast analysis speed, easy operation, and miniaturization, the chemiluminescence method has been widely used in biology, pharmacy, chemistry, environmental science, and clinical medicine [98,99,100]. Ma et al. [73] developed a chemiluminescent probe using an aptamer for detection of kanamycin at trace levels in aquatic products (Figure 4A). They used the chemiluminescent transition metal platinum (Pt) rather than a traditional organic dye because the optical physical properties of Pt were more sensitive to microenvironment changes, and it had a longer phosphorescence half-life and a larger Stoke shiftvalues. The chemiluminescent probe used a Pt complex for signal transduction. Normally, the chemiluminescence emitted by this complex in water was very weak, but when inserted into the double helix DNA structure, the strength of the chemiluminescence increased, which gave excellent signal transduction. When only the Pt complex and aptamer were present in water, the aptamer was in a free folded state. When kanamycin was added to the system, the aptamer specifically bound to it and its conformation changed to a hairpin structure. This double-helix hairpin structure promoted insertion of the Pt complex into the aptamer fragment and enhanced the chemiluminescence signal. The detection limit in an aqueous solution was 143 nmol/L and the linear range was 0.2–150 mol/L. Lin [74] developed a simple, rapid, and highly sensitive method for kanamycin analysis using carbon nanoparticles (CNPs) and an aptamer. In this method, luminescent CNPs with high water stability and excellent luminescence were synthesized by a microwave-assisted method (Figure 4B). Amine-modified kanamycin aptamer was fixed on the surface of the CNPs with carboxyl groups, which gave the CNPs aptasensor excellent selectivity and stability. Kanamycin was analyzed using the developed CNPs aptasensor on the basis of competitive inhibition mechanism. The content of kanamycin in milk was analyzed successfully with a detection limit as low as 5 × 10^−8^ ng/mL.

## 6. SPR Aptamer Sensors

With the SPR technique, compounds are detected using refractive index changes or chemical changes resulting from optical coupling of metal films (gold or silver). SPR is a highly sensitive optical sensing technology relying on the interactions of light with the free electrons in a semi-transparent noble metallic layer or chip and can realize the real-time monitoring of small changes in the effective refractive index of a metal-dielectric interface [101,102,103]. SPR sensors have the advantages of high throughput, no need for labeling, simple operation, and provide results faster than other methods. With the rapid development of aptamer technology, aptamer SPR biosensors have attracted increasing attention. An aptamer selected by the SELEX method can be fixed on a SPR chip surface. When the target object passes through the sensor chip, the aptamer connected to the SPR substrate will specifically recognize the target object, leading to changes in the resonance conditions, the reflectivity, and the SPR output signal for detection [104]. De-los-Santos-Alvarez [60] constructed a competitive aptasensor using methylated RNA aptamer-binding SPR technology and applied it to detection of neomycin B. SPR sensors provide sensitive and detailed information on the affinities and dynamics in biomolecular interactions. Real time binding curves can be obtained by monitoring the change in the angle of laser light on a gilded film. Both the concentration of antibiotics in the substance to be measured and the dissociation constant and stoichiometric value of neomycin B binding to its aptamer can be measured using the response of the SPR sensor. The linear range for detection of neomycin B in milk was 10 nmol/L–100 μmol/L and the detection limit was 5 nmol/L. Although SPR aptamer sensors have great potential in AGs testing due to the advantage of the high through analysis, the high cost of supporting equipment and chips limits its application in field testing. 

## 7. Electrochemical Aptasensors

Electrochemical aptasensors are modified antibiotic aptamers that use a substrate material with good conductivity. These types of sensors have become a focus in the field of antibiotic detection because they have good specificities, large linear ranges, low detection limits, low detection costs, are simple and fast to operate, are easy to miniaturize, and can be used for on-line monitoring. Nowadays, various electrochemical aptasensor designs have been established and extensively employed for applications related to clinical di-agnostics, biomedical research, environmental monitoring, and food analysis [105,106,107]. A typical electrochemical aptamer sensor consists of an electrode covered with an aptamer which, upon binding the analyte, undergoes a conformational switch affecting current flow through the system [57]. According to their output parameters, such as the impedance, current, and potential, electrochemical aptasensors can generally be divided into the following three types: impedance sensors, current sensors, and potential sensors [108]. 

**Figure 5:**
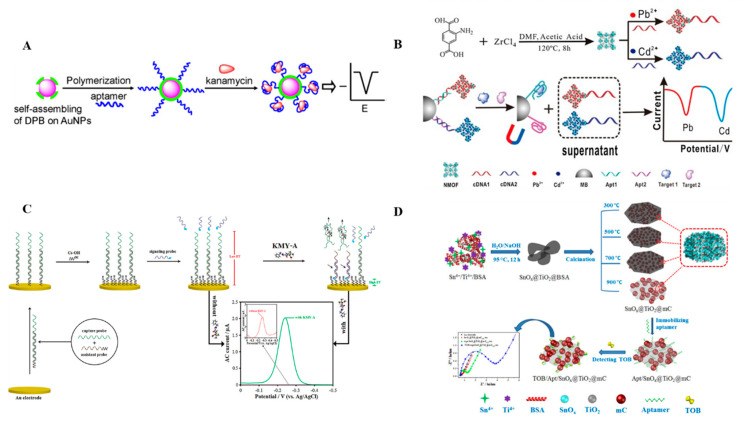
Schematic illustration of an electrochemical aptasensor. (**A**) Conductive polymer/gold self-assembled nanocomposite [76]. (**B**) Aptamer–metal ion nanoscale metal organic framework (MOF) electrochemical biocodes for detection of multiple antibiotics [78]. (**C**) The target-induced signal probe transfer mechanism [79]. (**D**) Preparation of BSA-directed SnO*_x_*@TiO_2_@mC nanocomposites and application to tobramycin detection [81].

Electrochemical impedance spectroscopy (EIS) displays impedance signals by monitoring changes in electron transfer resistance, whereas square wave voltammetry (SWV), differential pulse voltammetry, and alternating current voltammetry all show changes in current. In recent years, nanomaterials, such as carbon nanotubes, graphene, conducting polymers, and metal nanoparticles have been widely used to construct aptasensors because they have high specific surface areas, good biocompatibilities, high conductivities, unique physical and chemical properties, and excellent performance for improving the efficiency of electron transfer on an electrode surface [107]. Feng [75] prepared two kinds of electrochemical aptasensors by modifying the surfaces of glassy carbon electrodes with graphene and AuNPs by electrodeposition to use as carriers. The prepared aptasensors were applied to quantitative detection of kanamycin and streptomycin. Using differential pulse voltammetry, the detection limits for kanamycin and streptomycin were 0.03 pmol/L and 0.3 pmol/L, respectively. The sensor had high sensitivity, good selectivity, good reproducibility, and good stability. Zhu et al. [76] constructed an electrochemical aptasensor using a conductive polymer/gold self-assembled nanocomposite and applied it to detection of kanamycin with high sensitivity. They covalently immobilized a kanamycin aptamer onto a AuNP conductive polymer composed of poly-(2,5-di-(2-thienyl)-1H-pyrrole-1-(*p*-benzoic acid)) as a sensor probe (Figure 5A). The concentration of kanamycin was determined by voltammetry. The detection limit of the sensor was 9.4 ± 0.4 nmol/L. Xu et al. [77] used SWV with exonuclease in electrochemical sensors. Because of the cyclic shearing effect, the background signal was greatly compressed, which increased the absolute value of the change in the response signal and improved the sensitivity. Detection of kanamycin was realized with a detection limit as low as 1 pmol/L. Chen [78] proposed a new electrochemical biocode containing a nanoscale metal organic framework (NMOF) for simultaneous detection of multiple antibiotics, including kanamycin. In this study, an amine-functionalized NMOF was used as a substrate to carry different metal ions. The metal NMOFs were labeled with complementary strands of aptamers toward different targets. After specific binding of the aptamers to the targets, the corresponding metal NMOFs were released into the supernatant after magnetic separation and detected by SWV (Figure 5B). The method had high sensitivity and the detection limit was 0.16 pmol/L. Li et al. [79] constructed an electrochemical aptasensor using a target-induced signal probe transfer mechanism, and applied it to detection of kanamycin residues in milk, water, and serum samples using alternating current voltammetry (Figure 5C). This system gave an ultra-low background signal because of dissociation of the signal probe, which improved the sensitivity. The detection limit for kanamycin was as low as 3.3 pmol/L, and the detection was rapid. Sharma [80] developed an impedance aptasensor using a functionalized aptamer complementary sequence for a silk-screen-printed carbon electrode for the detection of kanamycin in milk using EIS. The signal mechanism of the sensor involved enhancement of the impedance. Before addition of the target compound, the electron transfer efficiency was high and the impedance was low. After addition of the target, the aptamer specifically captured the target and formed a complex covering the electrode surface. This obstructed the electron transfer channel and increased the impedance. The concentration of the target could be measured using the change in impedance. Under the optimized analysis conditions, the detection limit of the sensor was 0.11 ng/mL and the linear range was 1.2–75 ng/mL, which was far less than the residue limit for kanamycin in milk (150 ng/mL). Wang [81] constructed an electrochemical aptasensor using protein-oriented CNPs embedded with nanocrystalline SnO*_x_* and TiO_2_ to detect tobramycin with good sensitivity. A series of mesoporous carbon nanospheres embedded with SnO*_x_* and TiO_2_ nanocrystals were obtained by pyrolysis of a SnO*_x_*@TiO_2_@bovine serum albumin nanocomposite at different temperatures using titanium butyrate and sodium stannate trihydrate as precursors and bovine serum albumin as the template (Figure 5D). SnO*_x_*@TiO_2_@mC900 exhibited good electrochemical activity and high biological affinity among a series of SnO*_x_*@TiO_2_@mC nanocomposites. According to the electrochemical impedance spectroscopy results, the LOD was 6.7 pg/mL. The fabricated SnO*_x_*@TiO_2_@mC900 nanocomposite aptasensor had an ultra-low detection limit for tobramycin. A number of electrochemical aptasensors were introduced in the past years to improve performance and simplify the AGs detection on site, but the sensitivity of these aptasensors was not sufficient to apply them in real samples, as the problem of interface effect on electrode surface.

## 8. Other Aptasensors

In summary, many aptamer biosensors have been developed for the detection of AGs, but most of these are targeted to single compounds. Sensors that can detect groups of compounds are preferred for rapid on-site detection. Caglayan [109] designed an aptasensor-based elliptical polarized light sensor for the determination of AGs in dairy products. Kanamycin and neomycin were successfully detected with good sensitivity and minimum detection limits of 0.22 ng/mL and 0.048 ng/mL, respectively. Tang and his team [110] designed an evanescent wave sensor (EWA) using target binding to promote fluorescence quenching for group-specific detection of AGs in a fully online mode. In fluorescence quenching with an EWA, a fluorophore-labeled DNA aptamer with selectivity for kanamycin was used for both recognition of the target in solution and signal transduction to the EWA optical fibers. The number of the aptamers form multiple-strand complex (M-APT) on the fibers was inversely proportional to the AG concentration. The minimum detection limit of this method for AGs was 26 nmol/L. The sensor responded specifically to all AGs detected, but not to other types of antibiotics. With the development of digital technology, intelligent platforms are frequently used in scientific research. Using a digital fluorescence detector as readout device, Wang [111] developed an intelligent platform for detection of multiple AGs using a ratiometric paper-based device. Quantitative detection was realized according to the relationship between the change in the digital fluorescence detector signal and the target concentration. Sensitive analysis of streptomycin, tobramycin, and kanamycin could be realized simultaneously using this platform.

## 9. Conclusions

With improvements in production and living standards, environmental protection and food safety issues have aroused widespread concern. In recent years, the extensive use and even abuse of antibiotics have posed a serious threat to the environment and food safety. Consequently, detection of antibiotic residues in food and the environment has attracted increasing attention. With the rapid development of aptamer screening technologies, biosensors containing aptamers have provided a new method for rapid detection of AG residues in the environment and food. At the same time, various nanoscale and composite materials combined with electrochemical, optical, and photoelectrochemical detection technologies have been used to develop aptasensors with different signal amplification and output modes. Although there has been progress in research on biosensors developed from AG aptamers, most sensors are in theoretical and laboratory-research stages. Therefore, practical application of these sensors on a large scale remains distant. Consequently, it is important to develop rapid, high-quality, inexpensive, digital, and intelligent biosensor technologies. Future work should focus on the following aspects. First, there are few types of aptamers that can be used for the detection of AG residues. Screening for more types of antibiotics or class-specific aptamers that can recognize common groups will be important for detection of AG residues. Second, development of multi-functional nanomaterials and strengthening of the application compatibility between nanomaterials, molecular recognition elements, and conversion elements is required to improve aptasensors and meet the need for portable, inexpensive, and simple sensors that can be used for on-site testing. Third, environmental and food sample matrices are complex and high-throughput and specific sample purification and enrichment methods aptasensor are required to reduce the impact of matrix effects on aptasensors and improve the accuracy. With the rapid development of aptamers, problems restricting the development of sensors will eventually be overcome. The development of a fast, sensitive, and portable aptamer biosensor will broaden the application range and commercial prospects for rapid detection of AG residues in the field.

## Figures and Tables

**Table 1:** Maximum residue limits (MRLs) of some aminoglycosides in various countries.

Drug Name	Species or Product	Detection Object	MRL (μg kg^−1^)			
			China	The European Union	The United States	Japan
Gentamicin	Pig, Cattle	Muscle, Fat	100	50	100/400	100
		Liver	2000	200	300	2000
		Kidney	5000	750	400	5000
		Milk	200	100	-	200
	Chicken	Tissue	100	-	100	-
Kanamycin	Cattle	Muscle, Fat	-	100	-	40
		Liver	-	600	-	40
		Kidney	-	2500	-	40
		Milk	-	150	-	400
	Pig, Chicken	Muscle	-	100	-	100/50 (chicken)
		Fat	-	100	-	100
		Liver	-	600	-	100/50 (chicken)
		Kidney	-	2500	-	100/500 (chicken)
	Chicken	Egg	-	-	-	500
Neomycin	Cattle, Pig, Chicken	Muscle, Fat, Liver	500	500	1200/-/3600	500
		Kidney	10,000	5000	7200	10,000
		Milk	500	1500	150	500
		Egg	500	500	-	500
Streptomycin/Dihydrostreptomycin	Cattle, Sheep, Pig	Muscle, Fat, Liver	600	500	500	600
		Kidney	1000	1000	2000	1000
	Cattle	Milk	200	200	-	200

**Table 2:** Specific aptamer sequences of several AGs.

AGs	Aptamer Sequence (5′–3′)	*K*_d_ (μmol L^−1^)	Ref.
Kanamycin	TGGGGGTTGAGGCTAAGCCGA	0.079	[59]
Neomycin B	GGCCUGGGCGAGAAGUUUAGGCC	1.24	[60]
Streptomycin	TAGGGAATTCGTCGACGGATCCGGGGTCTGGTGTTCTGCTTTGTTCTGTCGGGTCGTCTGCAGGTCGACGCATGCGCCG	0.199	[61]
Tobramycin	GACTAGGCACTAGTC	0.042	[62]

**Table 3:** Summary of aptasensor types, detection methods, and performances.

Sensor Type	Method	Strategy	Analytes	LOD*	Ref.*
Colorimetric	NaCl-AuNPs	High-salt induce AuNPs* aggregation from red to blue	Kanamycin	25 nmol/L	[59]
		the catalytic chromogenic reaction of AuNPs mimics enzymes	Streptomycin	86 nmol/L	[65]
	NaCl-AgNPs	based on analyte-protected and aptamer-selective mechanism	Kanamycin	2.6 ng/mL	[66]
Fluorescent	Labeled	labeled with Exo III, AuNPs and FAM*.	Kanamycin	321 pmol/L	[67]
		Poly A and FAM modified at the two ends of aptamer	Neomycin B	0.01 μmol/L	[68]
		A fluorescent “signal-on” switch aptasensor based on QDs-SSB*	Streptomycin	0.03 ng/mL	[69]
		The fluorescence resonant energy transfer based on UCNPs* and graphene oxide (GO)	Kanamycin	9 pmol/L	[70]
	Non-labeled	digestion of dsDNA by ExoIII and the ability of SYBR Gold as a fluorescent dye	Streptomycin	54.5 nmol/L	[71]
		dsDNA-capped mesoporous silica nanoparticles and Rhodamine B	Kanamycin	7.5 nmol/L	[72]
Chemiluminescence	Cold light probe	The Pt complex performs signal transduction	Kanamycin	143 nmol/L	[73]
	FALIA* assay	CNP*-aptasensor probe	Kanamycin	5 × 10^−8^ ng/mL	[74]
Surface plasmon resonance	Competitive effect	RNA aptamer modified by methyl groups	Neomycin B	5 nmol/L	[60]
Electrochemical	Differential pulse voltammetry (DPV)	GR* and AuNPs modified on glassy carbon electrode surface as adaptor carriers	Kanamycin Streptomycin	0.03 pmol/L 0.3 pmol/L	[75]
		conductive polymer/gold self-assembled nanocomposite	Kanamycin	9.4 ± 0.4 nmol/L	[76]
	Square wave voltammetry (SWV)	The background signal compressed by exonuclease	Kanamycin	1 pmol/L	[77]
		nanoscale metal organic framework	Kanamycin	0.16 pmol/L	[78]
	Alternating current voltammetry (ACV)	Target induces signal probe transfer	Kanamycin	3.3 pmol/L	[79]
	Electrochemical impedance spectroscopy (EIS)	screen printing carbon electrode	Kanamycin	0.11 ng/mL	[80]
		protein-oriented carbon nanoparticles embedded with SnOx and TiO2 nanocrystalline	Tobramycin	6.7 pg mL^−1^	[81]

* Limit of detection(LOD), references (Ref.), Gold nanoparticles (AuNPs), fluorescein amidite(FAM), a single stranded DNA binding protein on quantum dots (QDs-SSB), upconversion nanoparticles (UCNPs), fluorescence-based aptamer-linked immunosorbent assay(FALIA), carbon nanoparticles(CNP), Graphene(GR).
